# Microseismic Time Delay Estimation Method Based on Continuous Wavelet

**DOI:** 10.3390/s22082845

**Published:** 2022-04-07

**Authors:** Cunpeng Du, Shengwen Yu, Haitao Yin, Zhen Sun

**Affiliations:** 1 College of Geodesy and Geomatics, Shandong University of Science and Technology, Qingdao 266510, China; ducunpeng@sdust.edu.cn; 2 Shandong Earthquake Agency, Jinan 250014, China; yinhaitao121@163.com; 3 College of Oceanography and Space Information, China University of Petroleum, Qingdao 266510, China; b20160007@s.upc.edu.cn

**Keywords:** microseismic signal, continuous wavelet transform, similarity coefficient method, cross-correlation, time delay estimation

## Abstract

The microseismic signal is easily affected by observation noise and the inaccurate estimation of traditional methods will seriously reduce the location accuracy of the microseismic event. Therefore, based on the continuous wavelet spectrum and the similarity coefficient, a fast and efficient microseismic time delay estimation method is proposed. Firstly, the original signals are denoised by continuous wavelet transform. Subsequently, the time-frequency transform of the original signal by continuous wavelet transform, time-frequency signal extraction is the process of band-pass filtering, which can further reduce the influence of noise interference on the time delay estimation. Finally, we calculated the similarity between the time-frequency signals via the time domain and frequency domain integration. The similarity function is based on correlation and proposed according to the time-frequency transformation provided by the phase spectrum to evaluate the similarity between two noisy signals. The time delay estimation is determined by searching for the similarity function peak. The experimental results show the precision and accuracy of the method over the cross-correlation method and generalized cross-correlation phase transformation method, especially when the signal-to-noise ratio is low. Therefore, a new time delay estimation method for non-stationary random signals is presented in this paper.

## 1. Introduction

Microseismic monitoring is an important means to obtain information on the regional geological structure, crustal rupture direction, and stress state. The location of microseismic events is an important technology for microseismic monitoring. Accurate earthquake location is of great significance to the study of the distribution and deep structure of seismogenic faults. In the location of microseismic events, the arrival time difference at different stations relative to the same earthquake event is picked up by the cross correlation technique to determine the source location, hence the time difference directly affects the accuracy of the location results [1,2,3,4,5,6].

The time difference of the same event received by each station is the time delay. The commonly used time delay estimation (TDE) methods include the cross-correlation (CC) method [7,8,9], generalized cross-correlation phase transformation (GCC-PHAT) method [10,11,12,13], generalized phase spectrum method [14], cross-power spectrum method [15] and adaptive filtering method [16]. CC method is the most basic TDE in the time domain according to the correlation degree of the two signals, easily affected by observation noise. To eliminate or reduce the influence of observation noise on the TDE of the CC method, in 1976 Knapp and Carter proposed a GCC-PHAT method based on the idea of filtering [13]. This method reduces the influence of noise on TDE by filtering the received signal, that is, weighting the signal in the frequency domain. Compared with the CC method, this method can become better TDE when the signal-to-noise ratio (SNR) is low. The above methods are based on the correlation coefficient to evaluate the degree of correlation between the two signals, when there is a weak correlation between the two signals, it may fail due to the influence of observation noise. In 1822, Fourier proposed the concept of the Fourier transform, which established the connection between the time domain and the frequency domain of the signal and extended the signal analysis from the time domain to the frequency domain [17]. However, Fourier cannot fully describe the characteristics of signal parameters changing with time, so it cannot satisfy the analysis of non-stationary signals. Wavelets are a highly effective method for analyzing noisy data and are useful for correlation analysis of non-stationary signals [18]. Incorporating the time-frequency analysis theory into the time-delay estimation theory is a hot direction in the research of time-delay estimation in recent years [19,20], which can improve the accuracy of time-delay estimation, reduce the amount of computation and improve the convergence speed in the applications of multipath delay and variable delay. Hilbert–Huang’s time-frequency transform is incorporated into the time-delay estimation algorithm to improve the GCC algorithm, which can reduce the influence of noise on the accuracy of time-delay estimation and solve the problem of time-delay estimation under the condition of a low signal-to-noise ratio [21]. However, the flying wings at both ends of the signal will appear in the process of empirical mode decomposition (EMD) decomposition [22], resulting in false modal function components, affecting the accuracy of time-delay estimation.

At present, the application of time-frequency analysis to the time-delay estimation algorithm is still an improvement in the CC method [23,24]. The time-delay is estimated through the correlation in the time domain or the frequency domain and can reduce the influence of background noise to a certain extent but has its limitations. For this reason, this paper focuses on the continuous wavelet spectrum based on the concept of the similarity coefficient (SC) proposed by Sun and Liu [25,26] and proposes a fast and efficient method for estimation of SC microseismic time delay based on continuous wavelet transform (CWT). The microseismic signal is decomposed and reconstructed, and then the similarity of the two microseismic signals is calculated to estimate the time delay, which has the advantages of high calculation accuracy and strong anti-noise performance.

## 2. TDE Algorithm

### 2.1. Cross-Correlation TDE Method

Cross-correlation is a basic method used to compare the similarity of two signals or functions in the time domain [9]. The basic idea of TDE is to use the cross-correlation function of two received signals $x_{1}\left(t\right)$ and $x_{2}\left(t\right)$ to calculate the time delay.

(1) $$x_{1}\left(t\right)=s\left(t\right)+n_{1}\left(t\right)$$

(2) $$x_{2}\left(t\right)=s\left(t-\tau_{d}\right)+n_{2}\left(t\right)$$

where, $x_{1}\left(t\right)$ and $x_{2}\left(t\right)$ are two independently received noisy seismic signals, s(t) is the original seismic signal, $n_{1}\left(t\right)$ and $n_{2}\left(t\right)$ are the noises of two signals, respectively, and the signal and noise are independent of each other, $\tau_{d}$ is the relative delay of the signal. The correlation function of the signal is:

(3) $$R_{x_{1}x_{2}}\left(\tau \right)=E\left[x_{1}\left(t\right)x_{1}\left(t+\tau \right)\right]=R_{ss}\left(\tau -\tau_{d}\right)+R_{sn_{1}}\left(\tau -\tau_{d}\right)+R_{sn_{2}}\left(\tau \right)+R_{n_{1}n_{2}}$$

where E[●] stands for mathematical expectation, $R_{ss}\left(\tau \right)$ represents the autocorrelation function of the source signal s(t), satisfies:

(4) $$R_{sn_{1}}\left(\tau -\tau_{d}\right)=0,R_{sn_{2}}\left(\tau \right)=0,R_{n_{1}n_{2}}\left(\tau \right)=0$$

In other words, the source signal and the noise are completely orthogonal, the Equation (4) can be simplified as follows:

(5) $$R_{x_{1}x_{2}}\left(\tau \right)=R_{ss}\left(\tau -\tau_{d}\right)$$

And the properties of the autocorrelation function:

(6) $$\left|R_{ss}\left(\tau -\tau_{d}\right)\right|\leq R_{ss}\left(0\right)$$

It can be seen that if and only when $\tau -\tau_{d}=0$, $R_{ss}\left(●\right)$ takes the maximum value, that is, the correlation between the two signals is the maximum. Therefore, the time value of this time is used to locate the time delay of the two signals. This algorithm is simple and fast, but it cannot consider the stability of TDE.

To eliminate or weaken the influence of observation noise on the TDE determined by the CC method, Knapp and Carter proposed GCC-PHAT based on filtering in 1976 [13]. The time delay is an estimation by using GCC-PHAT of two received signals based on the phase transformation weighting function. In this method, the received signals are pre-whitened by filtering the received signal in the frequency domain, to reduce the influence of noise on TDE.

In seismic signal processing, the peak value of $R_{x_{1}x_{2}}\left(\tau \right)$ is not obvious due to the influence of noise, which reduces the accuracy of TDE. To sharpen the peak value of $R_{x_{1}x_{2}}\left(\tau \right)$, firstly, according to the prior knowledge of signal and noise, the cross-power spectrum is weighted in the frequency domain to reduce the interference of noise. Finally, the GCC-PHAT function is obtained by Fourier transform:

(7) $$R_{x_{1}x_{2}}\left(\tau \right)=\int_{0}^{x}\varphi_{PHAT}\left(\omega \right)X_{1}\left(\omega \right)X_{2}^{*}\left(\omega \right)e^{-j\omega \tau }d\omega$$

where x is the length of the data. Where the frequency domain weighted function is:

(8) $$\varphi_{PHAT}\left(\omega \right)=\frac{1}{\left|G_{x_{1}x_{2}}\left(\omega \right)\right|}=\frac{1}{\left|X_{1}\left(\omega \right)X_{2}^{*}\left(\omega \right)\right|}$$

$G_{x_{1}x_{2}}\left(\omega \right)$ is the cross power spectral density of $X_{1}\left(\omega \right)$ and $X_{2}\left(\omega \right)$.

### 2.2. Time Delay Estimation of SC Method Based on CWT

#### 2.2.1. Continuous Wavelet Transform

The Fourier transform and Gabor transform are effective for the processing of stationary signals or short-term stationary line numbers [7,27,28]. However, in many scientific experiments and engineering measurements, non-stationary signals are common, that is, the amplitude, frequency, and phase characteristics of signals change continuously with time. For non-stationary signals, there needs to be a time window in the high band to provide more frequency information; in the low-frequency band, there needs to be a long time window to describe the overall behavior of the signal, that is, a flexible and rigid time window is needed [19,20,29]. Gabor transform window size and shape are fixed, and cannot meet the needs of multi-resolution analysis, CWT is generated to meet the needs of multi-resolution analysis. The CWT is defined as follows:

Suppose $\varphi \left(t\right)\in L^{2}\left(R\right)$, if the Fourier transform $\hat{\varphi }\left(\omega \right)$ of φ(t) satisfies the admissibility condition:

(9) $$C_{\varphi }=\int_{R}\frac{{\left|\hat{\varphi }\left(\omega \right)\right|}^{2}}{\omega }d\omega <\infty$$

Then φ(t) is called the wavelet generating function. The function family $\left\{\varphi_{a,b}\left(t\right)\right\}$ generated by the scaling and translation of the generating function φ(t) is called a wavelet, and the wavelet basis function is:

(10) $$\varphi_{a,b}\left(t\right)=\frac{1}{\sqrt{a}}\varphi \left(\frac{t-b}{a}\right),a\in R^{+},a\neq 0,b\in R$$

where a is the scale factor, and b is the translation factor.

The continuous wavelet of a function $f\left(t\right)\in L^{2}\left(R\right)$ is defined as:

(11) $$\Psi f\left(a,b\right)=\int_{R}f\left(t\right)\varphi_{a,b}^{*}\left(t\right)dt,a\in R^{+},a\neq 0,b\in R$$

The time width and frequency width of the time-frequency window of wavelet transform is aΔt and Δω/a, the center of the window is $\left(at_{0}+b,\omega_{0}/a\right)$. It can be seen that the size of the time-domain window and the frequency domain window of the wavelet change with the change of a. The size of the window area Δt×Δω is constant, that is, scale, time and resolution restrict each other. When scale a increases, the time window becomes wider and the frequency becomes narrower for low-frequency analysis; when scale a decreases, the time window becomes narrower and the frequency window becomes wider, which is suitable for high-frequency analysis. Therefore, the wavelet transform has the characteristics of multi-resolution and multi-frequency.

**Figure 1:**
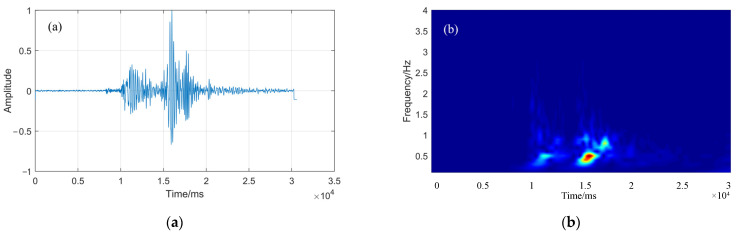
Microseismic waveform and time-frequency diagram. (**a**) the microseismic waveform diagram; (**b**) the time-frequency diagram.

When the signal-noise ratio is large enough, the CC method can determine the time delay by calculating the peak position of the cross-correlation function of the two signals. However, if there is background noise, the performance of the CC method will decrease sharply, so this paper uses CWT denoising and band-pass filtering to extract the useful components from the microseismic signal, to improve the accuracy of TDE. Figure 1 shows the microseismic signal waveform and continuous wavelet time-frequency diagram. It can be seen from Figure 1 that the original microseismic waveform data has a certain amount of background noise, which will affect the accuracy of TDE. After the time-frequency transformation of the original signal by CWT, the background noise in the signal can be better eliminated and the effective components of the microseismic signal can be extracted.

To evaluate the superiority of the SC method compared with the CC method and GCC-PHAT method in the correlation degree of two microseismic signals, the experiments add different SNR of Gaussian white noise to the microseismic signal, use three methods to calculate the time delay of the seismic signal and evaluate the result. CWT has a certain filtering ability and can obtain the time-frequency diagram of the seismic signal polluted by noise.

**Figure 2:**
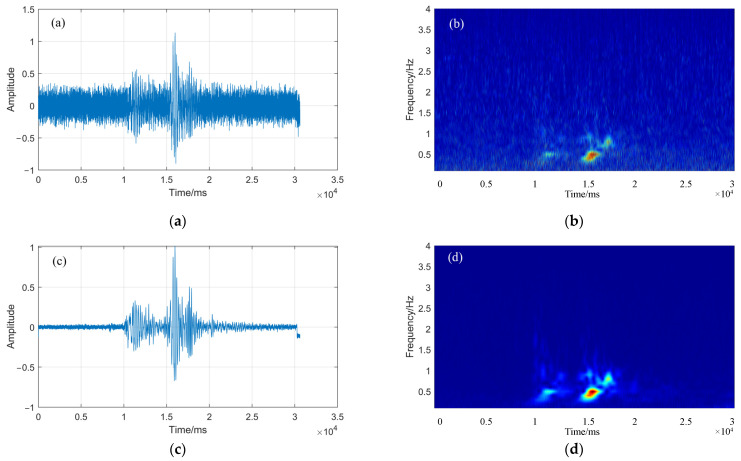
Seismic signals and time-frequency diagrams under different SNR (0 dB and 20 dB). (**a**,**b**) are the seismic waveform and time-frequency diagram of SNR = 0 dB; (**c**,**d**) are the seismic waveform and time-frequency diagram of SNR = 20 dB.

Figure 2 shows that the microseismic signal is the seismic waveform under the Gaussian white noise with an SNR of 0 dB and 20 dB, respectively, and the time-frequency diagram obtained by CWT. From the comparison between Figure 1a and Figure 2a, it can be seen that the noise with SNR of 0 dB has polluted the time domain and amplitude of the microseismic signal, changed the initial state of the seismic signal, and the added noise has covered part of the characteristics of the microseismic signal. Compared with Figure 1b, Figure 2b shows that there is obvious noise background on the time-frequency diagram after adding noise, and the microseismic signal area is still significant; compared with Figure 1a and Figure 2c, although the noise with SNR of 20 dB pollutes the noise of microseismic signal to a certain extent because the signal is filtered by CW, there is less noise background in the time-frequency diagram of Figure 2d, which indicates that the SC method can be used to estimate the time delay of the microseismic signal.

#### 2.2.2. Similarity Function

The similarity is used as a numerical index to measure the similarity of two signals [25,26]. The SC directly utilizes the instantaneous phases and instantaneous amplitudes of the time-frequency transform, not using the inverse transform. It is a direct method that can be used to judge the similarity between two signals and to estimate the time delay.

The similarity is based on correlation and proposed according to the time-frequency transformation provided by the phase spectrum to evaluate the similarity between two noisy signals. In terms of time delay estimation accuracy, due to its filtering function, similarity can determine the time delay of two signals more accurately and robustly than the correlation method, to determine the position of the signal source with high accuracy.

For two time signals $f_{1}\left(t\right)\in L_{1}\left(R\right)$ and $f_{2}\left(t\right)\in L_{2}\left(R\right)$, the time-frequency transform $\Psi f_{1}\left(\tau ,\varpi \right)$ and $\Psi f_{2}\left(\tau ,\varpi \right)$ of the two signals can be obtained according to the CWT. Then the similarity function is:

(12) ρ(d)=∫B∫DReΨf1(τ−d,ϖ)ReΨf2(τ,ϖ)dτdϖ∫B∫DReΨf1(τ−d,ϖ)2dτdϖ∫B∫DReΨf2(τ,ϖ)2dτdϖ

where Re denotes the real part, B denotes the frequency selection region, D denotes the time domain selection region, B and D are determined according to the prior information of time-frequency transformation, BD is the interest area that contains the signal in time-frequency, d is the shifting time index, ρ(d) is defined as the double integral of the time τ and frequency ϖ in the real part of the CWT spectrum function. When the conditions are met:

(13) $$\int_{D}\Psi f_{1}\left(\tau ,\varpi \right)d\varpi \approx \int_{D}\Psi f_{2}\left(\tau ,\varpi \right)d\tau \approx 0$$

The extreme value of ρ(d) is called the similarity between the two signals, the corresponding time is the delay time.

The Similarity is the correlation between the two signals after filtering. Since the actual signals are affected by noise, when the two microseismic signals are weakly correlated, the estimated delay will have a large deviation. The main difference between the SC, the GCC-PHAT and CC, is that the SC is defined in the time-frequency domain, while the latter two are calculated primarily in the time domain. The correlation coefficient can only represent the correlation of two signals in the time domain, while the similarity coefficient is calculated by using the time-frequency of the signal, which extends the correlation calculation to two-dimensions, which is more suitable for judging the correlation degree of the two signals.

## 3. Time Delay Estimation Experiments

### 3.1. The Correlation Coefficient of Time Delay Estimation

To accurately evaluate the superiority of the SC method in calculating the time delay, the true value of time delay between two microseismic signals is set to 10 in the experiment, and the maximum value of the correlation coefficient corresponds to the time difference between two signals, that is, the time delay value. Add different SNRs to the microseismic signal, and calculate its correlation coefficient and time delay value. Use the peak value and stability of the correlation coefficient to evaluate the three methods. The following tests will demonstrate that the SC method can effectively recognize similar signals compared to the GCC-PHAT and CC under high levels of background noise.

### 3.2. Accuracy of the TDE

To further verify the performance of the algorithm, different intensity random Gaussian white noise is added to the microseismic signal, and 100 repeated experiments are carried out under different SNR conditions to calculate the precision and accuracy of different methods. The SC is based on correlation and the similarity degree between two microseismic signals is evaluated according to the time-frequency transform of continuous wavelet, so the stability and accuracy of the time delay are analyzed mainly through the similarity and compared with the CC method and GCC-PHAT method. In this paper, the success rate (*SR*) and root mean square error (*RMSE*) are used to evaluate the precision and accuracy of TDE. The calculation formula for the *SR* is:

(14) $$SR=\frac{m}{n}\times 100\%$$

where m is the number of estimated values obtained by different TDE methods equal to the true time delay values, and n is the number of calculations at the same SNR, that is, n= 100. *RMSE* is used to evaluate the deviation between the estimated value and the true value of time delay between different methods, the calculation formula of *RMSE* is as follows:

(15) $$RMSE=\sqrt{\frac{1}{n}\sum_{i=1}^{n}{\left(x_{i}-\hat{x}\right)}^{2}}$$

where $x_{i}$ is the delay estimation value of different methods, $\hat{x}$ is the true value of the delay between the two signals.

## 4. Experimental Results and Analysis

**Figure 3:**
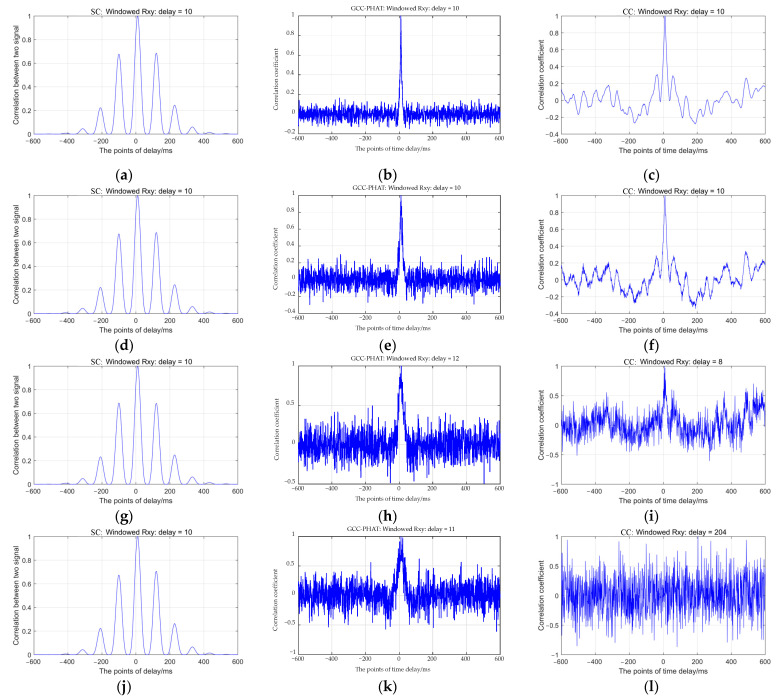
Time delay values calculated by the SC method: (**a**) SNR = 30 dB, delay = 10 ms, (**d**) SNR = 20 dB, delay = 10 ms, (**g**) SNR = 10 dB, delay = 10 ms, (**j**) SNR = 0 dB, delay = 10 ms; the GCC-PHAT method: (**b**) SNR = 30 dB, delay = 10 ms, (**e**) SNR = 20 dB, delay = 10 ms, (**h**) SNR = 10 dB, delay = 12 ms, (**k**) SNR = 0 dB, delay = 11 ms; and the CC method: (**c**) SNR = 30 dB, delay = 10 ms, (**f**) SNR = 20 dB, delay = 10 ms, (**i**) SNR = 10 dB, delay = 8 ms, (**l**) SNR = 0 dB, delay = 204 ms.

As can be seen from Figure 3, when the SNR is relatively large, the correlation coefficient peak value of the three methods is relatively obvious, and the estimation value of delay is relatively accurate, but with the decrease of the SNR and the increase of noise, there is a certain deviation in the time delay estimated by the GCC-PAHT method and the CC method. The time delay calculated by the SC method is better than the other methods under different SNRs, the peak value of the SC is obvious and the estimated delay value is 10. The SC method uses the CWT to filter the microseismic signal, which suppresses the influence of noise on the TDE, and this method is based on calculating the SC of two microseismic signals in the time domain and frequency domain, which can improve the stability of the TDE, has better anti-noise performance, can effectively process the noise in the signal, sharpen the amplitude and make the peak more prominent. The GCC-PHAT method filters the signal, that is, the frequency domain weighting processing, which can reduce the side lobe of the correlation coefficient, enhance the frequency components with high SNR in the signal, suppress noise, and improve the accuracy of the TDE algorithm. With the decrease in SNR, the peak value of the correlation coefficient of this method is still sharp, but the time delay has been affected by noise, and the result has a certain deviation. Because the traditional CC method itself does not have the function of signal filtering, the calculation result is greatly affected by noise when the SNR is low. There are many interference signals in the correlation coefficient calculated by this method, which makes the peak value not obvious, and the deviation of the TDE is large.

**Figure 4:**
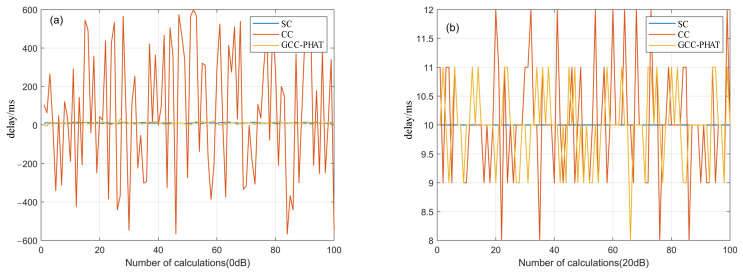
Time delay values calculated by three methods when the SNR is 0 dB (**a**) and 20 dB (**b**).

As seen in Figure 4a, when the SNR is 0 dB, the time delay deviation of the CC method reaches ±600 due to the influence of background noise, which has deviated from the true value. The time delay values of the GCC-PHAT method and the SC method are small, but there is still some deviation. As can be seen from Figure 4b, when the SNR is 20 dB, the time delay calculated by the SC method has no deviation, while the time delay value deviation of the CC method is ±2 and the GCC-PHAT method delay value is ±1. In contrast, the performance of the SC method is better and the CC method has lost its effect when the SNR is low.

**Figure 5:**
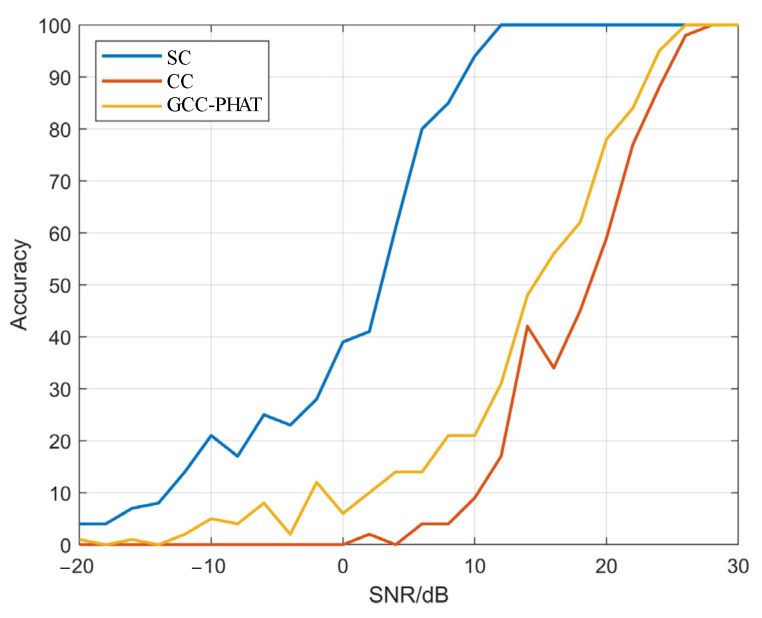
The accuracy of the calculation result of different SNR with three methods.

To further study the TDE accuracy of the three methods under different SNRs, Figure 5 shows the precision and accuracy of the three methods when the SNR is −10 dB to 30 dB. It can be seen that with the decrease of the SNR and the increase in noise, the precision and accuracy of the three methods are reduced to a certain extent. Among them, the accuracy of the CC method decreases the fastest, and combined with Figure 3l and Figure 4a, the CC method is greatly affected by noise because it does not filter the signal, and with the increase of SNR, the difference between the precision and the accuracy of the CC method and the GCC-PHAT method is getting smaller. The accuracy of the GCC-PHAT method is suddenly improved when the SNR is −6 dB, and the RMSE is 6.51, but the precision is low, is 0.08, which indicates that this method has certain limitations on the noise filtering of microseismic signals. When the SNR is low and the noise is higher than a certain degree, the performance of the algorithm degrades sharply, and the time delay cannot be estimated accurately.

**Figure 6:**
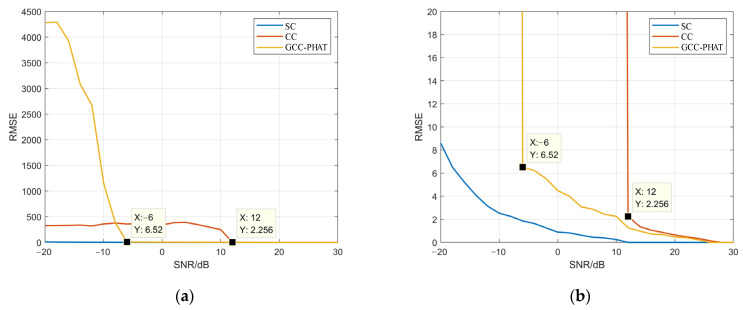
The precision of the three methods when the SNR is -20~30. (**a**) the RMSE of the TDE with different SNR; (**b**) the enlarged view of (**a**) with RMSE of 0–20.

The performance of the SC method is always better than the other two methods, especially when the SNR is low. When the SNR is 0dB, the precision of the SC method is 0.4, while the CC method and the GCC-PHAT method are less than 0.1. When the SNR is 12 dB, the TDE precision of the SC method is 1, while the precision of the GCC-PHAT method is 0.31, and the RMSE is 1.27. The precision of the CC method is 0.17, and the RMSE is 2.26. As can be seen from Figure 6a,b, the SC method has higher accuracy and robustness than the other two methods, mainly because the SC method has the filtering function and can reduce the influence ode noise on the microseismic signals to a certain extent, which prove the superiority of the SC method based on the CWT in microseismic signal TDE.

## 5. Conclusions

The microseismic signals have non-stationary characteristics, and the noise in the signal will affect the accuracy of TDE. CWT is used to denoise the signal to improve the estimation accuracy; the SC method is used to extend the correlation method in the time domain to two-dimensional TDE to improve the recognition rate of the microseismic signals. In the aspect of microseismic TDE, the SC method can estimate the time delay of two microseismic signals more accurately than the correlation method and obtain the source location with high precision. The correlation between the two microseismic signals when the SNR is low is difficult to show with the CC method and the GCC-PHAT method due to the influence of noise; both the accuracy rate and the accuracy are low. While the SC method itself has the filtering function, it can more accurately express the degree of similarity between the two signals, and the TDE results and accuracy are higher. The experimental results suggest that the method proposed in this paper is superior to the reference method.
